# Artificial Intelligence and Precision Health Through Lenses of Ethics and Social Determinants of Health: Protocol for a State-of-the-Art Literature Review

**DOI:** 10.2196/40565

**Published:** 2023-01-24

**Authors:** Sarah Wamala-Andersson, Matt X Richardson, Sara Landerdahl Stridsberg, Jillian Ryan, Felix Sukums, Yong-Shian Goh

**Affiliations:** 1 Department of Health and Welfare Technology, School of Health, Care and Social Welfare, Malardalen University Eskilstuna Sweden; 2 University Library, Malardalen University Eskilstuna Sweden; 3 School of Medical and Health Sciences, Edith Cowan University Perth Australia; 4 Directorate of Information and Communication Technology, Muhimbili University of Health & Allied Sciences Dar es Salaam United Republic of Tanzania; 5 Alice Lee Centre for Nursing Studies, National University of Singapore Singapore Singapore

**Keywords:** artificial intelligence, clinical outcome, detection, diagnosis, diagnostic, disease management, ethical framework, ethical, ethics, health outcome, health promotion, literature review, patient centered, person centered, precision health, precision medicine, prevention, review methodology, search strategy, social determinant

## Abstract

**Background:**

Precision health is a rapidly developing field, largely driven by the development of artificial intelligence (AI)–related solutions. AI facilitates complex analysis of numerous health data risk assessment, early detection of disease, and initiation of timely preventative health interventions that can be highly tailored to the individual. Despite such promise, ethical concerns arising from the rapid development and use of AI-related technologies have led to development of national and international frameworks to address responsible use of AI.

**Objective:**

We aimed to address research gaps and provide new knowledge regarding (1) examples of existing AI applications and what role they play regarding precision health, (2) what salient features can be used to categorize them, (3) what evidence exists for their effects on precision health outcomes, (4) how do these AI applications comply with established ethical and responsible framework, and (5) how these AI applications address equity and social determinants of health (SDOH).

**Methods:**

This protocol delineates a state-of-the-art literature review of novel AI-based applications in precision health. Published and unpublished studies were retrieved from 6 electronic databases. Articles included in this study were from the inception of the databases to January 2023. The review will encompass applications that use AI as a primary or supporting system or method when primarily applied for precision health purposes in human populations. It includes any geographical location or setting, including the internet, community-based, and acute or clinical settings, reporting clinical, behavioral, and psychosocial outcomes, including detection-, diagnosis-, promotion-, prevention-, management-, and treatment-related outcomes.

**Results:**

This is step 1 toward a full state-of-the-art literature review with data analyses, results, and discussion of findings, which will also be published. The anticipated consequences on equity from the perspective of SDOH will be analyzed. Keyword cluster relationships and analyses will be visualized to indicate which research foci are leading the development of the field and where research gaps exist. Results will be presented based on the data analysis plan that includes primary analyses, visualization of sources, and secondary analyses. Implications for future research and person-centered public health will be discussed.

**Conclusions:**

Results from the review will potentially guide the continued development of AI applications, future research in reducing the knowledge gaps, and improvement of practice related to precision health. New insights regarding examples of existing AI applications, their salient features, their role regarding precision health, and the existing evidence that exists for their effects on precision health outcomes will be demonstrated. Additionally, a demonstration of how existing AI applications address equity and SDOH and comply with established ethical and responsible frameworks will be provided.

**International Registered Report Identifier (IRRID):**

PRR1-10.2196/40565

## Introduction

### Overview

Precision health is described as the ability to assess disease risks at an individual level, detect early preclinical conditions, and initiate preventive strategies [1]. Precision health refers to personalized health care based on a person’s unique genetic, genomic, or omics composition within the context of lifestyle, social, economic, cultural, and environmental influences to help individuals achieve well-being and optimal health [2]. As a field of research, it is still being delineated in relation to other fields such as precision medicine [3], while at the same time giving rise to other research concepts that it may actually encompass, such as precision public health [4]. In this manner, the scope of precision health is broader than that of precision medicine, by including strategies for health promotion and disease prevention that occur outside the setting of health care institutions, which can be widely adopted [5]. Thus, precision health strives to match promotion, prevention, diagnostic, and treatment interventions with genetic, biological, environmental, and social and behavioral determinants of health in a more person-centered manner [6]. Precision health, in a nutshell, entails shifting the focus from treatment to promoting health and preventing the onset of diseases.

Precision health, an evolution of precision medicine, combines genetic and genomic sequence, protein, metabolite, and microbiome information collectively known as “omics” with lifestyle, social, economic, cultural, and environmental factors [1].

Precision health applications often combine more than one data source to assess risks and diagnose and initiate preventative interventions, including biological, genetic, environmental, social, and lifestyle-related measurements or observations. Although combining clinical- and community-based health data sources has not yet been established as necessary to fall within the realm of precision health, the field does provide the theoretical basis to amalgamate precision medicine and the social determinants of health (SDOH) [7] within the same interventions. This ambitious approach to health includes dynamic linkages between the research and practice of medicine, economics, population health, and public health [6]. 

Digital technologies such as artificial intelligence (AI) are playing an increasing role in precision health. AI systems are partially or wholly autonomous machine-based systems that can, using statistical and mathematical modeling techniques for a given set of human-defined objectives, make predictions, recommendations, or decisions influencing real or digital environments [8]. A recent review has identified few studies on machine learning techniques that promote behavioral changes and physical or mental health [9]. AI-based health technologies are rapidly entering the precision health domain outside the clinical setting. Examples of such technologies include screening and early detection devices and applications, health tracking applications, and mobile devices that support healthy living and improve chronic diseases management. In many cases, such technologies are integrated into devices where precision health was not the primary function or purpose. This may affect both the reliability and accuracy of, but also access to, precision health strategies. Some scholars have highlighted the potential of AI-based interventions in integrating the SDOH and precision medicine to improve overall population health [6,10].

With the prevalence and rapid development of health AI applications, the World Health Organization (WHO) has released a set of guiding principles for the ethical use and governance of AI in health [11]. Per the WHO guidelines, emphasis should be on (1) protecting autonomy; (2) promoting human well-being, human safety, and the public interest; (3) ensuring transparency, explainability, and intelligibility; (4) fostering responsibility and accountability; (5) ensuring inclusiveness and equity; and (6) being responsive and sustainable during its deployment. These guidelines thereby address a recent discussion of AI’s bias [12] and other challenges [13] to its use in health applications. The source of such bias and related challenges may be largely related to a long-entrenched health research focus on specific socioeconomic and demographic populations. A recent review further demonstrated that only 20% of studies include individuals from socioeconomically disadvantaged backgrounds [14]. There is, therefore, considerable risk for bias which should be clearly considered in AI algorithms that steer precision health interventions. 

The Organisation for Economic Co-operation and Development has also published 5 complementary values--based principles for the responsible stewardship of trustworthy AI [8]. Three Organisation for Economic Co-operation and Development countries’ health regulatory agencies further elaborated upon these values by providing a common set of machine learning best practices for medical device development [15]. A potential challenge with value-based ethical frameworks is their openness to interpretation and lack of enforceability. Health AI technologies’ rapid development and focus on individualization may also affect the longevity and scope of guidance for their application, suggesting a need for more tailored assessment than current standards [16], if not stricter regulations. The European Union (EU) Commission has also proposed a legal framework for trustworthy AI based on EU values and fundamental rights [17], stressing human-centrism, trust, safety and compliance with the law, and fundamental rights.

Several state-of-the-art reviews have been conducted for a broad array of AI-based applications in precision medicine [18], including oncological treatment [19], cardiovascular imaging [20,21], and optimization of clinical workflows [22]. However, similar, timely reviews of AI-based applications within the broader context of precision health are not yet available, and the types and categories of such applications are largely undefined. One summary of state-of-the-art consumer health informatics and education was conducted in 2018 [23] to elucidate opportunities, challenges, and practical implications of AI in these applications, stating that direction and evidence on the benefits of AI to individuals’ health were lacking. Since then, the global AI in health care market size is projected to grow from US $8.23 billion in 2020 to US $194.14 billion in 2030, growing at a rate of 38.1% [24]. 

### Review Questions and Objectives 

We plan to conduct a state-of-the-art literature review to address research gaps and reduce the knowledge gap regarding novel AI-based applications in precision health. The research questions guiding the review are as follows: 

- What novel AI-based applications exist that target precision health and what is their role? 
- How are AI-based applications categorized? 
- What evidence exists for the effects of AI application on precision health outcomes? 
- How do AI-based applications account for ethical and responsible AI principles according to the recent WHO and other guidelines, and if so, how are they operationalized?
- How do AI-based applications for precision health address socioeconomic determinants of health? 

The objectives for the review are therefore: 

- to identify and categorize novel precision health-related AI applications reported in both scientific and gray literature, 
- to summarize the most recent evidence demonstrating effects of AI-based interventions in contributing to precision health outcomes,  
- to identify evidence for compliance of AI-based applications in precision health with the WHO principles for ethical AI according to the WHO [11], and 
- to assess how AI-based applications for precision health affect health equity in relation to socioeconomic determinants of health [25].  

## Methods

### Overview

This article outlines the protocol for an original state-of-the-art review that will be undertaken and reported according to PRISMA (Preferred Reporting Items for Systematic Reviews and Meta-Analyses) [26]. State-of-the-art reviews address more current matters and the most current literature in contrast to other kinds of reviews, offering new perspectives on the issue in focus and highlight areas in need of further research [27,28]. 

The review will encompass applications that use AI as a primary or supporting system or method when primarily applied for precision health purposes in human populations. Any geographical location or setting, including web-based, community-based, and acute, or clinical settings will be included, reporting clinical, behavioral, and psychosocial outcomes, including detection-, diagnosis-, promotion-, prevention-, management-, and treatment-related outcomes. Primary empirical research studies published in scientific or gray literature in the English language will be included for all dates prior to onset of searches.

Textbox 1 describes the planned eligibility criteria for the review based on population, intervention, contexts, and types of studies or evidence. 

**Eligibility criteria for the planned state-of-the-art review.**

**Inclusion criteria**

- Human participants of any age, health status, residence and background; and their samples, material, or data related to their health
  *Note: The use of samples, material, or data falls within the inclusion criteria. Fictional or fabricated material will be included as long as the trained models are validated for external previously unseen real populations.*N
- Applications that use artificial intelligence (AI) as a primary or supporting system or method, when primarily applied [1] for precision health purposes
- Any geographical location or setting, including web-based, community-based, acute, or clinical settings
- Clinical, behavioral, and psychosocial outcomes, including detection-, diagnosis-, promotion-, prevention-, management-, and treatment-related outcomes
- Primary empirical research studies published in scientific or gray literature (see information sources)
- English language for scientific and gray literature; those that can be obtained as full texts or reports
- All dates prior to the onset of searches

**Exclusion criteria**

- Any participants, samples, material, or data not obtained or derived from humans
- AI-based systems or methods that are primarily applied for other reasons, even if they indirectly affect precision health outcomes
*Note: An example of an excluded intervention would be an AI-based application for tailored marketing of services or products, which affects the users’ psychological or well-being status.*
- Economic or organizational outcomes (eg, cost-effectiveness or organizational efficiency studies)
- Qualitative, nonempirical studies, editorial or opinion pieces, or reviews
- All other languages; articles that cannot be obtained as full texts or reports

### Information Sources 

Scientific database searches will be conducted from all dates prior to the onset of searches in Medline (EBSCOHost), CINAHL Plus (EBSCOHost), APA PsycInfo (EBSCOHost), Scopus [29], Web of Science [30], IEEE [31], and Cochrane Central Registry of Controlled Trials [32].

Searches in gray literature, or “that which is produced on all levels of government, academics, business and industry in print and electronic formats, but which is not controlled by commercial publishers, i.e., where publishing is not the primary activity of the producing body” [33], will be conducted. The purpose of the gray literature searches is to identify ongoing or completed studies that are not available in the commercial scientific literature databases. Links to such studies may also be found in device registries. The following gray literature sources will therefore be searched: 

- Study registries and registers of medical devices: 
  WHO ICTRP [34]
  ClinicalTrials.gov [35]
  FDA list of approved AI-enabled medical devices [36]
  EU EUDAMED register [37]
  International Health Technology Assessment Database [38]
  arXiv (stat.ML, cs.AI, cs.LG) [39]
  medRxiv [40]
- Multidisciplinary gray literature databases: 
  OAlste [41]
  Bielefeld Academic Search Engine [42]
  Dissertations and thesis databases: 
  Dissertations and Theses A&I (ProQuest) 
  Open Access Theses and Dissertations [43]
  DART-Europé [44]
- Web search engines:
  Google scholar [45]

### Search Strategy 

The preliminary search terms and number of retrieved documents are provided in Table 1. The full review will include both free-text and controlled vocabulary, and the search terms will be adapted for each database. The search terms related to the intervention precision health through AI will be divided into 2 blocks. Proximity operators will be used, where possible, to increase the chance of finding relevant references. Searches of gray literature will be simplified to individual terms if required. No date limit will be applied.

**Table 1:** Example of the search strategy in Medline (EbscoHost, October 17, 2022)

Search #	Search blocks	Retrieved documents, n
1	TI ( (genomic* OR precis* OR personali* OR individuali* OR stratif* OR tailo* OR adapt* OR custom* OR predictive) N2 health* ) OR AB ( (genomic* OR precis* OR personali* OR individuali* OR stratif* OR tailo* OR adapt* OR custom* OR predictive) N2 health* )	18,454
2	(TI “Artificial Intelligence” OR AB “Artificial Intelligence”) OR (TI “machine learning” OR AB “machine learning”) OR (TI “deep learning” OR AB “deep learning”)	104,534
3	(MH “Artificial Intelligence+”)	156,342
4	2 OR 3	208,639
5	4 AND 1	571
Limiters	Narrow by Language: English	566

A larger alteration concerning the search block relating to AI is applied based on the recent publication [46]. However, due to complexity, databases such as IEEE and Cochrane Trials are excluded. Yin et al [46] argue that AI is a broadly encompassing term and includes specific AI techniques, such as neural networks, support vector machines, decision trees, and natural language processing. Yet, such studies are highly likely to use “artificial intelligence” or “machine learning” in abstracts or keywords [47].

The final search terms and eligibility criteria (Textbox 1) will be designed to capture a range of precision health research. The search strings are presented in Table 1.

### Data Management Plan 

All identified records will be entered into EndNote (Version X20, Clarivate Analytics) and duplicates will be removed. Remaining records will then be entered into and managed within the Covidence systematic review software (Veritas Health Innovation Ltd) for the remaining review process.

The PRISMA guidelines for reporting of systematic reviews [27] will be followed. Three researchers will conduct the review process in 4 steps:

1. Initial screening: the titles, keywords, and in some cases abstracts of the obtained records will be screened for relevance by 2 reviewers independently in duplicate and voted upon for relevance for further review. Consensus will result in inclusion or exclusion, with conflicts resolved by a third researcher.
2. Full-text screening: the full text for all studies proceeding to this step will be obtained and read independently by 2 reviewers, who will vote upon inclusion in the next step. Consensus will result in inclusion or exclusion, with conflicts resolved by the third researcher. 
3. Citation searching: the cited references for, and citations of, any studies included after the full-text screening step will be searched in the same manner as the source articles, and then follow the steps preceding this in the review process. 
4. Data extraction: essential information regarding the study aim, design, conduct, population, intervention, and outcomes, as well as results data for relevant outcomes, will be extracted from each included study by 2 reviewers independently. Consensus will result in the inclusion of the extracted data in the review’s summary of findings, with conflicts resolved through dialogue or, failing consensus thereafter, by the third researcher. 

### Quality Assessment of AI Interventions

Due to the diversity and heterogeneity of existing AI techniques, significant biases are likely to occur, making it difficult to interpret the results and implement these models in practice [48]. AI-based models rely substantially on data, quality, quantity, and type of data to ensure high algorithmic accuracy. Additionally, studies related to AI interventions require high methodological quality and rigorous standards of outcome reporting and representative study participants.

Thus, Quality Assessment of Diagnostic Accuracy Studies 2 (QUADAS-2) will be used to validate the quality of studies in the review [49]. The QUADAS-2 tool includes 4 domains covering flow and timing, reference, standard, and patient selection. Each domain is evaluated for biases and assessed for applicability.

### Framework for SDOH

There is no universal standardized method for categorizing SDOH. For example, the WHO’s conceptual framework includes micro-, meso-, and macrolevel measures [50]. SDOH is further defined by the Centers for Disease Control and Prevention as “conditions in the environments in which people are born, live, learn, work, play, worship, and age that affect a wide range of health, functioning, and quality-of-life outcomes and risks” [51]. Thus, SDOH falls into 5 key categories: health care access and quality, education access and quality, social and community context, economic stability and the neighborhood, and the built environment.

For this review, the framework for SDOH will be based on the sociocultural environment within the framework for digital health equity. This includes social determinants that include downstream and upstream factors at the individual, interpersonal, community, and societal levels [52].

### Ethics and Dissemination  

This study will not involve human participants and their primary data or unpublished secondary data, thus ethical approval is not required. Findings of the state-of-the-art review will be disseminated through publication in an open-access scientific journal as well as through research networks and journalistic media. 

### Patient and Public Involvement

The primary and secondary analyses will be presented to representatives of multiple stakeholder groups, including users or patients, professionals, and public purchasers (if relevant). Their input will then be solicited to assist in the secondary analyses regarding anticipated impact and consequences on equity and the SDOH. 

## Results

### Overview

This article is step 1 toward a full state-of-the-art literature review with data analyses, results and discussion of findings which will also be published.

The anticipated consequences on equity from the perspective of SDOH will be analyzed. Keyword cluster relationships and data analyses will be visualized to indicate which research foci are leading the development of the field and where research gaps exist. Results will be presented based on the data analysis plan that include primary analyses, visualization of sources, and secondary analyses. Results from this review will potentially guide continued application and development of AI applications and pinpoint agenda for future research to reduce the knowledge gaps and improve practice related to precision health. New insights regarding examples of existing AI applications, their categorization and role regarding precision health, and the existing evidence for their effects on precision health outcomes will be demonstrated. Additionally, how existing AI-based applications account for ethical and responsible AI principles and address socioeconomic determinants of health will be analyzed.

### Primary Analyses 

The main statistics in each study relevant to the aims of the review will be reported as well as grouped across studies by target population (eg, older individuals, risk groups, disease state), channel (eg, device, software, or app), setting (eg, home or primary care), area of focus (eg, detection and treatment), and outcome (clinical, behavioral, and psychosocial). Depending on the statistical reporting of the studies, which is often less rigorous in gray literature, further synthesis may be conducted. As this is not a systematic review, the strength of the cumulative body of evidence will not be assessed.  

### Secondary Analyses 

The compliance of the precision health AI interventions or applications with the WHO principles for ethical AI [11] will be assessed and analyzed on quantitative (number of principles) and qualitative (degree or veracity of fulfilment of principles) levels. The anticipated consequences on equity from the perspective of SDOH will be analyzed.

## Discussion

### Overview

This article is step 1 toward a full state-of-the-art literature review with data analyses, results, and discussion of findings, which will also be published.

Keyword cluster relationships and data analyses will be visualized to indicate which research foci are leading the development of the field and where research gaps exist. Results will be presented based on the data analysis plan that include primary analyses, visualization of sources, and secondary analyses. Results from this review will potentially guide continued application and development of AI applications and pinpoint agenda for future research to reduce the knowledge gaps and improve practice related to precision health. New insights regarding examples of existing AI applications, their categorization, and role regarding precision health and the existing evidence for their effects on precision health outcomes will be demonstrated. Additionally, how existing AI-based applications account for ethical and responsible AI principles and address socioeconomic determinants of health will be analyzed.

This protocol is step 1 in the full state-of-the-art review that intend to provide insights on the evidence-based novel AI applications that promote precision health and how these applications comply with the WHO principles for the ethical use and how they affect health equity in relation to socioeconomic determinants of health.

As per our knowledge, this will be the first review addressing and precision health through lenses of ethics and SDOH. As an example of the search we conducted, search strategy in Medline (EbscoHost, October 17, 2022) indicated a total of 566 retrieved documents. Updated searches will be conducted in the full review.

Primary analyses will include main statistics in each study relevant to the aims of the review as well as grouped across studies by target population, setting, area of focus, and outcome. Visualization of sources will include mapping the existing literature on the use of AI in precision health and will help illustrate what precision health-related AI applications exist.  

Secondary analyses will include compliance of the precision health AI interventions or applications with the WHO principles for ethical AI and consequences on equity. Due to the diversity and heterogeneity of existing AI techniques, and the significant biases that are likely to occur, QUADAS-2 will be used to validate quality of studies in the review [49]. This will enable interpretation of the results from the review and address implementation of AI models in practice [48].

### Conclusions

This protocol will guide the development of the full review article. Results will potentially guide continued development of AI applications, future research in reducing the knowledge gaps, and improve practice related to precision health. New insights regarding examples of existing AI applications, their categorization and their role regarding precision health, and the existing evidence exists for their effects on precision health outcomes will be demonstrated. Additionally, demonstration of how existing AI applications address equity and SDOH and comply with established ethical and responsible framework will be provided.

Implications for future research and person-centered public health will be discussed in the full review in the light of strengths and limitations.

## Abbreviations

AI: artificial intelligence
EU: European Union
PRISMA: Preferred Reporting Items for Systematic Reviews and Meta-Analyses
QUADAS-2: Quality Assessment of Diagnostic Accuracy Studies 2
SDOH: social determinants of health
WHO: World Health Organization
